# Year-round hourly temperature and humidity sensor readings from arid caves, Judean Desert, Israel

**DOI:** 10.1038/s41597-025-06420-8

**Published:** 2025-12-17

**Authors:** Micka Ullman, Mitya Kletzerman, Asaf Oron, Roi Porat, Boaz Langford, Amos Frumkin, Yitzchak Jaffe, Uri Davidovich, Nimrod Marom

**Affiliations:** 1 School of Archaeology and Maritime Cultures, University of Haifa, Haifa, 3498838 Israel; 2 Conservation Department, Tel Aviv Museum of Art, Sderot Sha’ul HaMelech 27, Tel Aviv-Jaffa, Israel; 3 Institute of Earth Sciences and the Israel Cave Research Center (ICRC), The Hebrew University of Jerusalem, Jerusalem, 9190401 Israel; 4 Geological Survey of Israel, Jerusalem, 9692100 Israel; 5 Institute of Archaeology, The Hebrew University of Jerusalem, Jerusalem, 9190501 Israel; 6 Associated Researcher, ICArEHB, University of Algarve, Portugal

**Keywords:** Ecology, Environmental sciences

## Abstract

Monitoring microclimatic conditions in underground environments is crucial for understanding chemical and biological processes occurring in caves and their effect on archaeological, palaeontological, and palaeobotanical records. The Israel Cave Climate Project (ICCP) dataset provides high-resolution microclimatic data from twelve caves across three climate zones — Desert, Steppe, and Mediterranean — measured during 2019–2021 using a uniform protocol. All twelve are natural karstic caves containing diverse, rich, and typically multi-period archaeological records. Within each cave, hourly air temperature and relative humidity measurements were recorded over a year, and these data are presented here in full. The physical and speleological characteristics of the studied caves and the content and nature of their archaeological records are also detailed. The combined high-resolution datasets, incorporating speleological, climatological, and archaeological records, provide unparalleled raw data valuable for studying cave environments, particularly cave archaeology, site formation processes, and preservation and conservation of ancient material and bioarchaeological records.

## Background & Summary

Monitoring microclimatic conditions is fundamental in various fields of cave research (*Speleology*), having important implications for the study of cave formation processes, ecology and biology^1–4^. In the field of cave archaeology and material-culture conservation, monitoring microclimatic parameters in underground environments is crucial for understanding past and present formation processes and their effects on the preservation of ancient materials. Such monitoring is commonly conducted as part of efforts to protect and restore cave art^5–7^, but fewer such endeavors are dedicated to cave sites containing other types of extraordinary, sensitive finds. The Israel Cave Climate Project (ICCP) was specifically designed to address the exceptional preservation of archaeological and palaeontological finds observed in hundreds of natural caves in a specific arid region—Judean Desert—on the background of the environmentally-fragmented southern Levant.

**Fig. 1:**
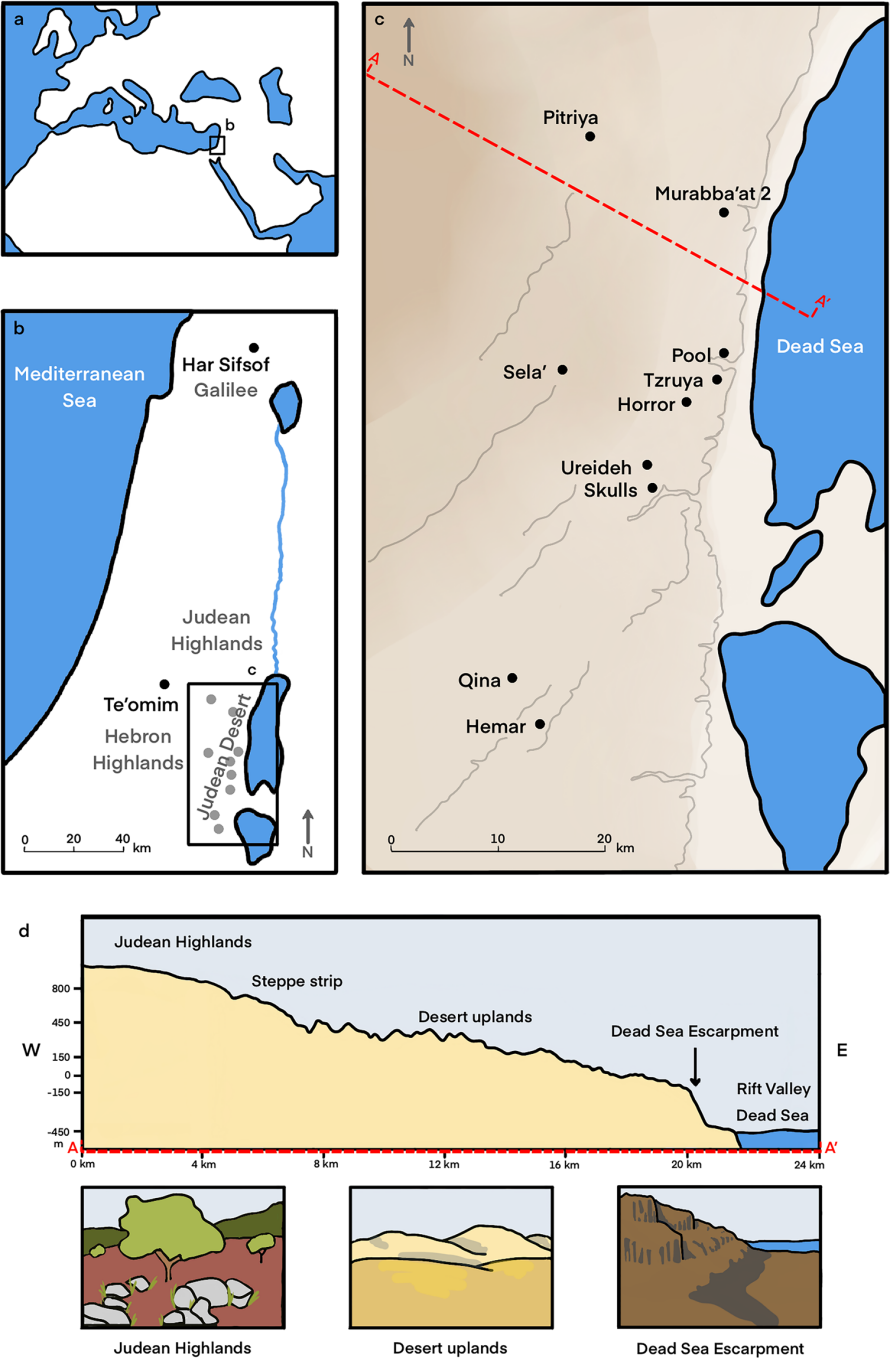
(**a**) A general location of the research area; (**b**) geographic location of the Judean Desert and the two caves sampled in the Mediterranean Climate Zone, Har Sifsof (Galilee) and Te’omim (western Judea Highlands); (**c**) the location of the ten caves sampled in the Judean Desert. The location of the topographic section A-A’ (**d**) is marked by a dashed red line; (**d**) a West-East topographic section of the Judean Desert (Illustration by A. Marck).

The ICCP datasets provide hourly temperature and humidity readings from data loggers placed in different locations within ten caves situated in the dry, arid environment of the Judean Desert, and two caves (Te’omim and Har Sifsof) located in the Mediterranean region of the Levant, serving as ex-regional control (Fig. 1; Table 1). Conditions in Mediterranean-zone caves differ markedly from the microclimatic conditions measured in the desert caves, with moist environments leading to poor preservation of organic and other materials in the former caves. The datasets detail the caves’ speleological and physical characteristics and the cave locations where the microclimatic data were recorded. The archaeological and ecological records of the caves are specified to the level of find categories (e.g., pottery, osteological finds, vegetal remains), serving as a proxy for the environmental preservation conditions that prevailed on site. The archaeological record in the studied caves covers various segments of the rich Holocene history of the Levant.

**Table 1:** The studied caves and their geographical location, arranged from north to south.

#	Cave Name	Region	Climate zone	Longitude	Latitude	Elevation (m)
1	Har Sifsof	Galilee	Mediterranean	35.42647	33.00885	806 asl
2	Te’omim	Judean Highlands	Mediterranean	35.02063	31.72621	386 asl
3	Pitriya	Judean Desert	Steppe	35.23969	31.65085	506 asl
4	Murabba’at 2	Judean Desert	Desert	35.37189	31.58849	20 asl
5	Pool	Judean Desert	Desert	35.37327	31.47286	10 bsl
6	Sela’	Judean Desert	Steppe	35.21438	31.45639	580 asl
7	Tzruya	Judean Desert	Desert	35.36449	31.44961	130 asl
8	Horror	Judean Desert	Desert	35.33322	31.4316	170 asl
9	Ureideh	Judean Desert	Desert	35.2932	31.37256	300 asl
10	Skulls	Judean Desert	Desert	35.30401	31.35993	230 asl
11	Qina	Judean Desert	Desert	35.16886	31.19403	425 asl
12	Hemar	Judean Desert	Desert	35.19384	31.15921	195 asl

Location coordinates given in the WGS-84 grid.

### Environment and climate

The Judean Desert is a rain-shadow desert^8^, formed east of the 800–1000 m asl Judean Highlands, as a result of the steep topographic gradient towards the Dead Sea Rift Valley, ca. 400 m bsl (Fig. 1). The Judean Highlands are dominated by Mediterranean climate, whereas a narrow strip in their eastern flank is typified by Steppe, semi-arid climate; aridity increases further east, with desert conditions prevailing^9^. In January, the mean temperature is 10 °C in the Judean Highlands and 17 °C in the Dead Sea Rift Valley, while in July it is 25 °C and 36 °C, respectively. Mean annual precipitation is 600–500 mm in the Judean Highlands, 400-300 in the Steppe strip, 200-100 in the Desert uplands, and 100–50 mm in the Rift Valley (Israel Meteorological Service, 2025). In both regions, natural caves formed during the Late Cenozoic were extensively used in various prehistoric and historical periods^10,11^. Notably, despite their location in a harsh desert environment away from major settlements, the Judean Desert caves were utilized by humans in antiquity for various purposes, including herding, ritual seclusion and caching, and refuge^10,12–17^.

**Fig. 2:**
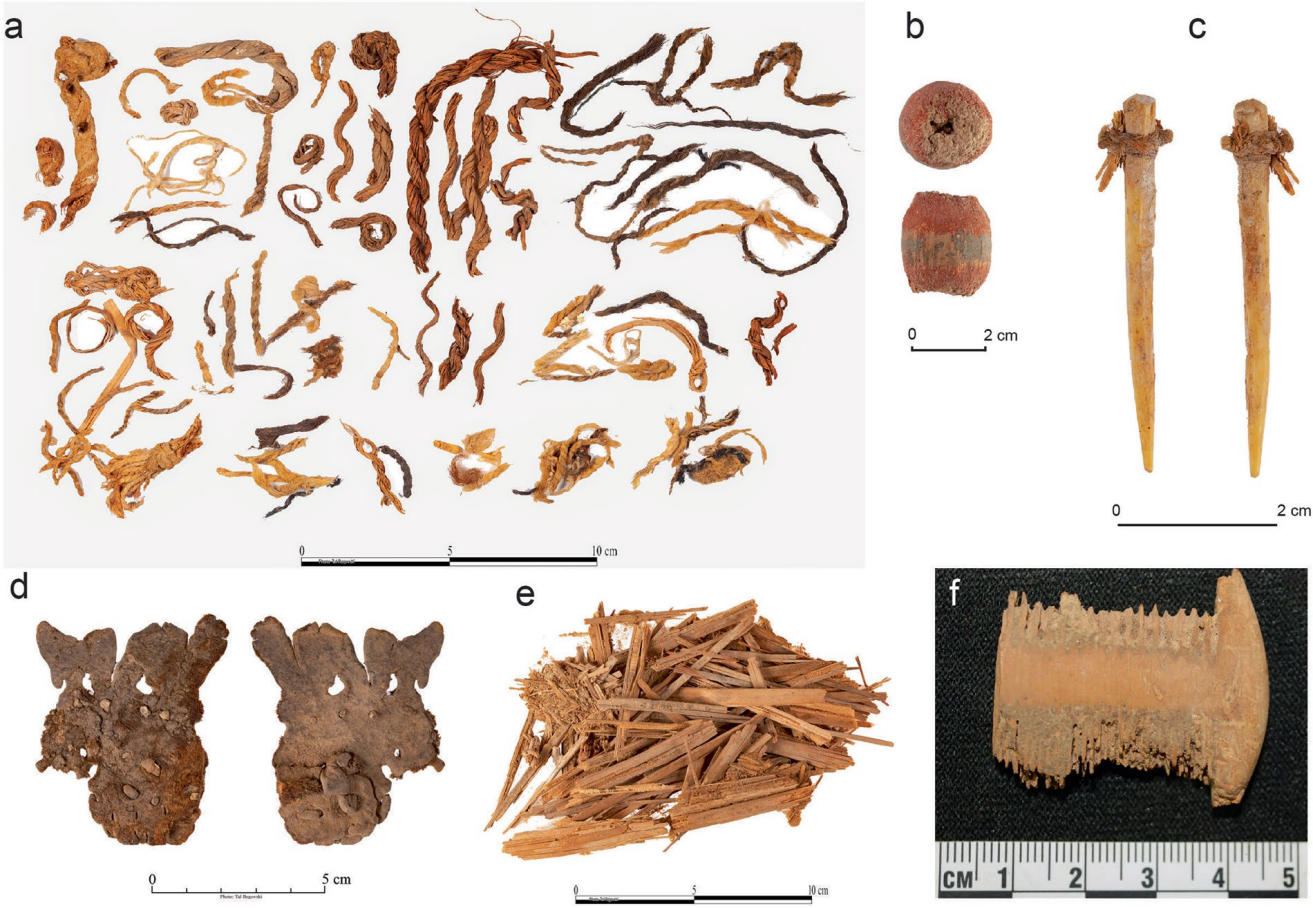
Various organic materials found in the Judean Desert caves: (**a**) ropes and basketry items made of vegetal materials, leather, or tendons, various periods; (**b**) a wooden bead, Pre-Pottery Neolithic B; (**c**) a bone point with an attached cord made of hide, Late Chalcolithic period; (**d**) a sandal made of hide, Roman period; (**e**) grasses used for matting, Late Chalcolithic period; (**f**) a wooden comb, Roman period (photographs by T. Rogovsky).

The extreme aridity of the Judean Desert caves enables exceptional preservation of ancient artifacts and ecofacts (Fig. 2). The Judean Desert caves have been known for over a century as a repository of cultural treasures, especially organic remains dating from the 10^th^ millennium BP onwards, often found in pristine conditions. These include the famous Dead Sea Scrolls, inscribed on animal hides and papyri and dated from the late first millennium BCE and the early first millennium CE^14,18^; the Late Chalcolithic hoard of hundreds of elaborate metal items, accompanied by various organic materials, found in the Cave of the Treasure in Nahal Mishmar and dated to the second half of fifth millennium BCE^19^; and the outstanding finds, including figurines, decorated human skulls, stone masks, basketry and textile items and numerous other finds from Nahal Hemar Cave, dated to the Pre-Pottery Neolithic B, ca. ninth millennium BCE^20^ (Table 2). Recently, a palaeoecological project focusing on the faunal record from these and other Judean Desert caves uncovered similarly rare preservation of late Pleistocene animal remains. Furthermore, while collagen preservation was generally poor, specific remains produced the oldest DNA sequences to have ever been recovered in Israel^21–23^. In addition, Judean Desert caves yielded numerous macro-botanical remains in pristine dry state from multiple periods, including domesticated cereals with the oldest complete DNA sequences^24,25^. Although the caves are known for their excellent preservation conditions and outstanding ancient material records, no systematic, high-resolution data collection of microclimatic measurements was previously conducted to clarify the specific conditions of cave environments.

**Table 2:** Types of finds and represented periods of human activity in the studied caves.

Cave		Har Sifsof	Te’omim	Pitriya	Murabba’at 2	Pool	Sela'	Tzruya	Horror	Ureideh	Skulls	Qina	Hemar
Artifacts made of inorganic materials	Groundstone (limestone, basalt)	+	+		+	+	+	+	+	+	+	+	+
	Flint	+	+	+	+	+	+	+	+	+	+	+	+
	Bead (minerals)	+	+		+	+	+	+	+	+	+		+
	Pottery	+	+	+	+	+	+	+	+	+	+	+	+
	Metal (mainly copper- and iron-based)	+	+	+	+	+	+	+	+		+	+	
	Glass		+		+	+	+		+		+		
Artifacts and ecofacts made of organic materials preserved in most archaeological contexts	Charred botanical remains	+	+		+	+		+	+	+	+	+	+
	Shell	+	+				+	+		+	+	+	+
	Skeletal remains (human, fauna)	+	+	+	+	+	+	+	+	+	+	+	+
	Bone, ivory	+	+		+	+		+	+		+	+	+
Organic artifacts and ecofacts preserved under special conditions	Wood, reed (uncharred)				+	+		+	+	+	+	+	+
	Seed, fruit (uncharred)				+	+		+	+	+	+		+
	Rope, basketry				+	+		+	+		+	+	+
	Papyrus				+	+			+		+		
	Textile				+	+		+	+		+	+	+
	Hide, skin				+	+		+	+		+	+	+
	Hair							+	+		+		
Occupation periods	Epi-Paleolithic (23,000–11,500 BP)				?		+			+			
	Pre-Pottery Neolithic (9500–6500 BCE)				?	+	+			+			+
	Pottery Neolithic (6500–4500 BCE)	+	+		+	+			?				
	Late Chalcolithic (4500–3700 BCE)		+	+	+	+	+	+	+	+	+	+	?
	Early Bronze Age (3700–2450 BCE)		+	+	+	+	+		+	+	+	+	+
	Intermediate Bronze Age (2450–1950 BCE)		+	+									
	Middle and Late Bronze Age (1950–1140 BCE)		+		+		+				+		
	Iron Age (1140–586 BCE)		+		+	+	+						
	Persian-Hellenistic (586–63 BCE)		+		+		?						
	Roman-Byzantine (63 BCE–628 CE)	+	+	+	+	+	+		+	+	+	+	
	Islamic (628–1917 CE)		+		+		?					+	

A “?” indicates insufficient data for precise dating.

Worldwide, monitoring cave microclimates has become widespread since the second half of the 20^th^ century, as part of various speleological studies. Commonly recorded parameters are air temperature, humidity, CO_2_ partial pressure, Radon concentration, and airflow velocity. A great improvement in parameter recording was achieved in the last decades, mainly due to new technologies, particularly the introduction of relatively inexpensive data loggers, which record a large amount of data^1^. Long-term cave microclimate monitoring has been carried out worldwide^3,26–28^. Despite the Levant’s rich cave environments, high-resolution datasets are scarce^29^. Current knowledge mostly relies on sporadic, manually collected measurements during short-term research visits^30^.

The current research presents raw, high-resolution datasets from twelve caves located in three different climate zones in the southern Levant, which were measured over a similar period of time using a uniform protocol. The study focuses on the Judean Desert caves, where, despite the importance of this region for world archaeology, environmental cave monitoring was never conducted. Furthermore, measurements in dry desert caves enable the recording of changes in air humidity, which in the commonly-studied active karstic caves cannot be efficiently recorded due to technical limitations, i.e., logger saturation, a phenomenon in which the logger fails to accurately record humidity fluctuation after reaching a reading of 100% humidity^1,3^.

## Methods

Water content and air temperature are key variables affecting the preservation quality of archaeological artifacts and ecofacts. Water may be present as liquid or vapor, and, combined with air temperature, influences a broad spectrum of physical, chemical, and biological processes and dictates material preservation quality and the rates of mechanical and chemical deterioration^31–33^. The geophysical characteristics and climate of the Judean Desert govern the microclimatic conditions within the caves. Due to the overall aridity and the regional hydrological regime, water is rarely present in liquid form. Thus, the impact on the deterioration of organic materials within the caves is affected by the amount of water vapor, which is evaluated as Relative Humidity (RH, %), the variable that measures relative air saturation with water vapor under a given temperature. This variable is calculated as the percentage ratio between Absolute Humidity (AH, g/m^3^), i.e., the actual quantity of water in the air at a given temperature, with its Saturation Humidity value (SH, g/m^3^), i.e., the maximum quantity of water the air could hold at this temperature before condensation occurs: ${RH}=\frac{{AH}}{{SH}}{\rm{\times }}100$. An RH of 100% indicates that the air is saturated, whilst an RH of 0% indicates a complete absence of water vapor. If a volume of moist air is enclosed in a sealed space, the AH remains constant, while the RH varies due to changes in air temperature: as the temperature drops, SH drops, and the RH rises.

To characterize the hygrothermal profiles of the Judean Desert caves, we designed a sampling methodology for measuring air temperature and relative humidity in a series of caves in various locations throughout the region. Similar measurements were taken in two caves located in the Mediterranean climate zone for comparative analysis.

### The caves

**Fig. 3:**
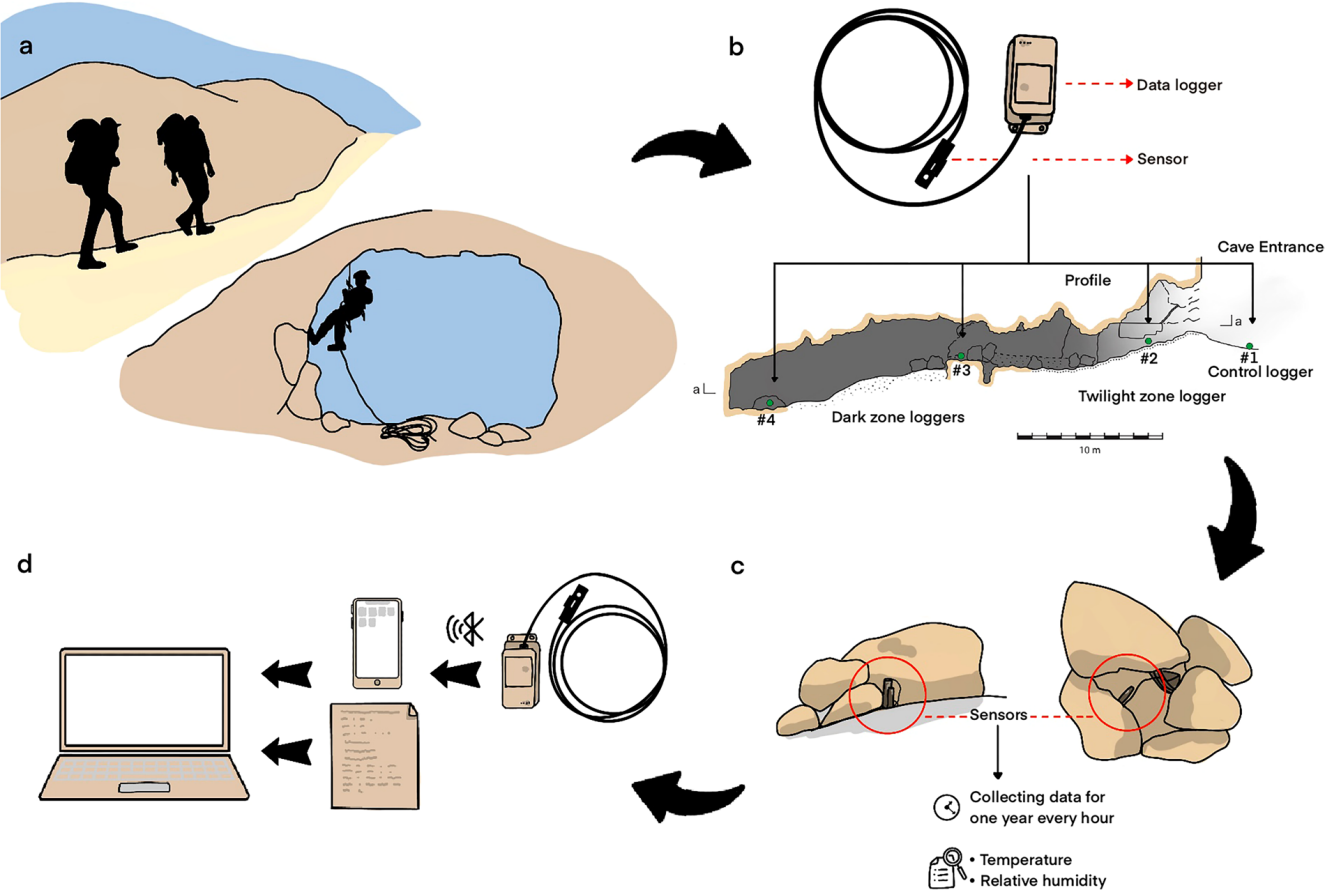
Workflow of loggers placement and documentation: (**a**) approaching the caves; (**b–c**) placement of loggers throughout the cave, marked on a cave profile (**b**) and shown in close-up (**c**); (**d**) transmission of recorded data from the logger to a mobile phone equipped with the HOBOmobile application, and compiling data and field notes into a computerized database (illustration by A. Marck).

The study took place in twelve caves (Figs. 1, 3; Table 1), selected on the basis of two main considerations. First, we aimed to sample cave sites that contain rich archaeological assemblages, particularly of well-dated organic materials, so that the relationship between recorded micro-environmental conditions and the degree of preservation could be inspected (Table 2). Second, caves were selected along both west-east and north-south climatic gradients, in order to account for inter-regional environmental variation. Among the twelve caves that were sampled, eight caves are located in the eastern flank of the Judean Desert, an area characterized by arid to hyper-arid climate; two caves are located at the western fringes of the Judean Desert, an area characterized by semi-arid Steppe climate; and two caves are located beyond the geographical boundaries of the Judean Desert, in Mediterranean climate, one in the western Judean Highlands and another in the Upper Galilee.

The studied caves are of hypogenic origin and are now considered relict karst features. Speleogenesis occurred during the Late Cenozoic, primarily during the Oligocene-early Miocene, after which the caves were uplifted above the water table. Following water table drop, the caves were modified by vadose processes such as ceiling collapse, speleothem deposition and sedimentation. Cave openings formed through surface denudation, subsequently enabling human and animal to access these subterranean systems^34–36^. Currently, these relict karst systems are either dry or experience limited drip water, affected by present meteoric hydrology and local subaerial topography^10,37–39^.

The fieldwork was coordinated with the Israel Nature and Park Authority. All but one of the studied caves (Har Sifsof) are located within nature reserves away from hiking trails. The approach to the caves was denied in winter and spring (November–April), during bats’ hibernation and roosting seasons. Particularly, the approach to cliff caves of the Judean Desert was denied throughout most of the year since these locations are favorable for the nesting of rare birds of prey. Therefore, only specific time slots were available for us to conduct fieldwork. Approaching the caves involved considerable physical effort and, at times, the use of specialized caving gear; it combined crossing rough terrains, scrambling over rocky cliffs, and negotiating various obstacles on the surface and within the caves.

### The data loggers

**Fig. 4:**
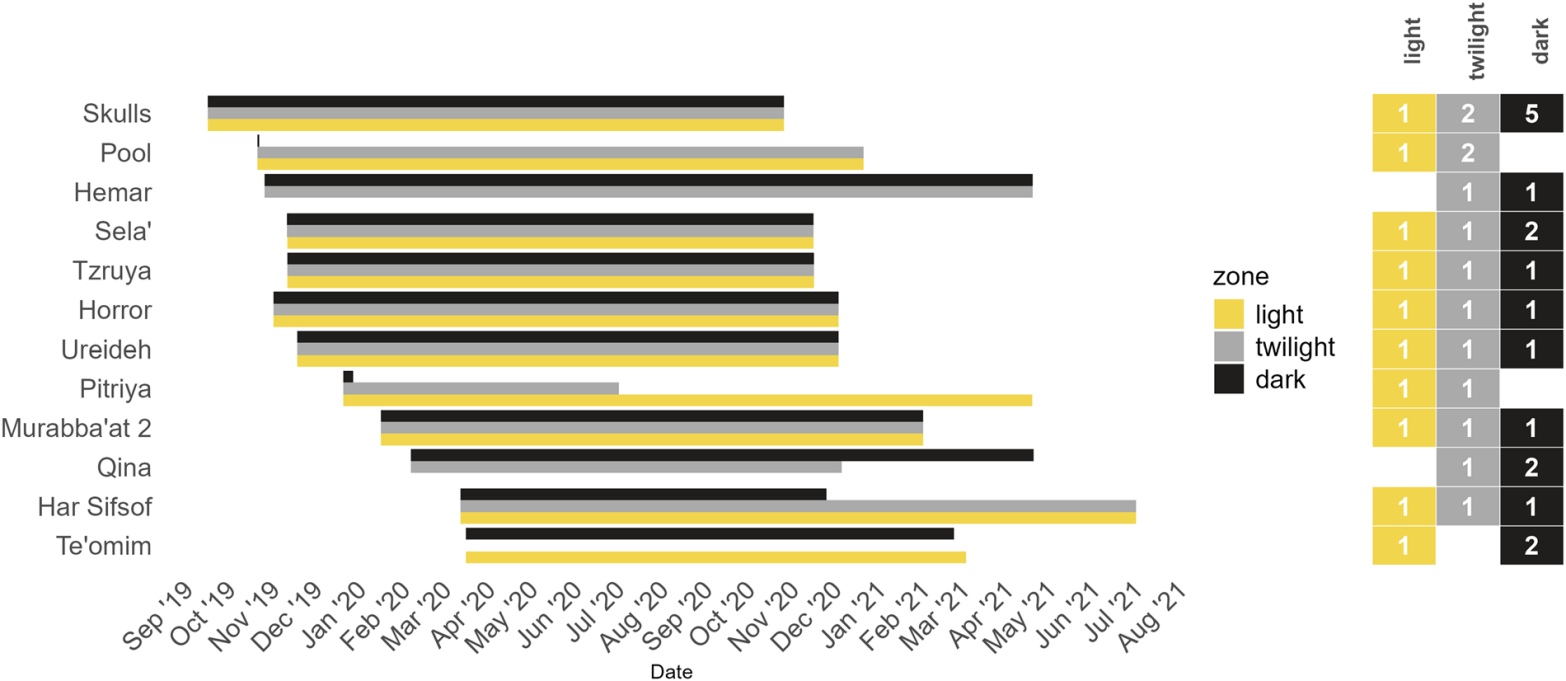
Data collection duration for each of the 12 sampled caves is presented by month and light zone. The number of loggers placed in each light zone of each cave is shown in the columns on the right.

The data loggers used in this research are of HOBO® ONSET® model MX2302A. Each logger has a temperature sensor and a relative humidity sensor (Table 3). The loggers were programmed to record measurements at an interval of 1 hour by means of the proprietary application HOBOmobile v-s. 1.9–2.0, via Bluetooth. It took us longer than 12 months to return to several caves and collect the loggers; as a result, the recording period was somewhat longer than one year. In other caves, loggers ceased recording before their due date (Fig. 4; and see below).

**Table 3:** HOBO MX2302A External Temperature/RH Sensor Data Logger, technical information.

Temperature Sensor	
**Range**	External temperature sensor: −40 to 70 °C
**Accuracy**	±0.25 °C from −40 to 0 °C ±0.2 °C from 0 to 70 °C
**Resolution**	0.02 °C
**Drift**	<0.01 °C per year
**Relative Humidity Sensor**	
**Range**	0 to 100% RH, −40 to 70 °C; exposure to conditions below −20 °C or above 95% RH may temporarily increase the maximum RH sensor error by an additional 1%
**Accuracy**	±2.5% from 10% to 90% (typical) to a maximum of ±3.5% including hysteresis at 25 °C; below 10% RH and above 90% RH ±5% typical
**Resolution**	0.01%
**Drift**	< 1% per year

### Strategy of logger positioning

A minimum of three loggers were installed for each cave: one in a shady spot just outside the entrance to record ‘control’ surface data (labelled as “light zone”, Figs. 3, 4), and two or more within the subterranean space. Depending on the cave’s extent and the availability of suitable installation spots, we aimed to place several loggers under different light conditions within the cave, which we termed “dark” and “twilight” zones. The dark zone typically comprises one of the innermost segments of the cave, while the twilight zone is a transitional area between the cave entrance and the deeper, darker parts. In several cases, more than one logger was placed in the dark and/or twilight zones, especially in sizable caves, where conditions may vary between various cave segments. It is important to note that although Judean Desert caves are remote and difficult to access, they are visited occasionally by hikers, shepherds and antiquities looters, so loggers had to be as invisible as possible, and camouflaged.

### Data collection workflow

1. Before going out to the field, loggers were wirelessly set up using Bluetooth and the *HOBOmobile* application within Android mobile platform.
2. Each logger was marked with a paper sticker and permanent marker, stating the cave name and logger number. For example, four loggers were placed in the Pool Cave, and thus, they were labeled: Pool Cave #1–4 (Fig. 3b).
3. The loggers were placed so that only the sensor is exposed to the open air, while the logger unit was hidden in a small niche or under a pile of stones. The sensor was stabilized using a small wooden stick and plastic binders to prevent it from touching the cave floor or walls (Fig. 3c). In several cases, the loggers were placed at an elevated point, and the sensor was left hanging in the air.
4. The loggers were turned on minutes before being placed. Once a logger was installed, the *HOBOmobile* application was used to ensure it was properly programmed and working.
5. The logger’s precise location and serial number were documented on the cave map and by photography (Fig. 3b). The location of each cave was determined according to a coordinate point taken at the center of the cave entrance using a manual Garmin *eTrex* device, with an accuracy of ±5 m. The cave was mapped using standard speleological methods^40^ with an accuracy of approximately 0.1 m. The positions of the sensors were marked on the cave maps based on visual identification of the cave’s morphology, with an estimated accuracy of ±0.25 m.
6. The installation date and time, as well as context information (cave name, logger number and serial number, and precise place of positioning within the cave) were documented in a field notebook.
7. Once placed in the cave or adjacent to its entrance, the loggers were left untouched for at least 12 months and occasionally longer.
8. Upon returning to the cave to collect the loggers, the collection date and time were recorded in the field notebook.
9. The loggers’ operation was stopped upon its collection, or later that day, in the lab.
10. Data was downloaded from loggers via Bluetooth, by the *HOBOmobile* Android Application, and saved in CSV format.

### Data compilation

The datasets from all the loggers, in addition to logger and cave metadata, were gathered within a netCDF file built in R v. 4.5.2^41^ and the libraries *RNetCDF*^42^, *lubridate*^43^, *tidyr*^44^, *dplyr*^45^, and *sf*^46^. The netCDF file was built following the conventions *ACDD-1.3* (Attribute Convention for Dataset Discovery) and *CF-1.8* (Climate and Forecasting metadata conventions); the dataset also complies with *FAIR* principles^47^.

### Input (comparative) Data

Mean daily temperature and precipitation over a specified observation period for each cave location were taken from the *CHELSA* (Climatologies at High Resolution for the Earth’s Land Surface Areas) project^48,49^. We retrieved these data using *Rchelsa* library^50^. However, as of the time of submission, precipitation data from 2021 onward was not yet fully available.

Since retrieving these data is time-consuming, we have also provided it as a standalone CSV file which comes with our *ICCP* observer package^51^. Updated data can be obtained either separately or by the means of *getChelsaData* script provided within the package.

## Data Records

The dataset is available at CERN’s Data Centre *Zenodo*^51^, with this section being the primary source of information on the availability and content of the data being described. The data file is accompanied with 55 cave photos and 12 cave maps (schemas) which are provided as a standalone archive; both are accessible on-line from *Zenodo*^51^. Cave maps consist of planar views, profiles and cross sections. On each map the position of the loggers and their sequential number (#1, #2 etc.) are indicated. The symbology used in these maps is presented in Frumkin 2015^10^.

Additionally, we provide a data viewer developed under *Shiny* framework^52^ for use in the *R* environment. The *ICCP* package allows observing the ICCP and CHELSA data in a friendly interactive way; the source code accompanied with the documentation, installation and executing manuals is also available on-line via *Zenodo*^51^.

The netCDF dataset file follows a multidimensional structure typical for climatic data. The file has three major **dimensions**: *time*, *cave*, and *logger*; additional dimension *reference* was added to keep the references of the previous cave studies. The **variables** inside the netCDF file are fully attributed and can be classified to three types: identifiers or mapping variables (“time”, “cave”, “logger”, “reference”, “Logger_Number_in_Cave”, “cave_references”, and “logger_cave_mapping”), data variables (“Temperature”, “Relative_Humidity”, and “Dew_Point”), and metadata variables—the majority of all the variables—whose names and descriptions we list here:

- **RefCitation**: references to previous research related to the site;
- **Cave_Name**: the cave name in English;
- **Region**: geographical region of cave location;
- **Longitude**: cave’s main entrance longitude (WGS84) [degrees east];
- **Latitude**: cave’s main entrance latitude (WGS84) [degrees north];
- **Elevation**: elevation of the cave main entrance above the sea level [meters];
- **Total_Length**: aggregated length of all cave sections measured during cave mapping [meters];
- **Topography**: topography of surface adjacent to cave;
- **Phytogeographic_Region**;
- **Main_Entrance_Width**: maximal width of the cave’s main entrance [meters];
- **Main_Entrance_Height**: maximal height of the cave’s main entrance [meters];
- **Secondary_Entrance_Width**: maximal width of the cave’s secondary entrance, if present [meters];
- **Secondary_Entrance_Height**: maximal height of the cave’s secondary entrance, if present [meters];
- **Lithostratigraphy**;
- **Cave_Formation**: major speleological and deformation processes that formed the cave’s physical characteristics;
- **Current_Environment**: current microclimate environment inside a cave;
- **Structure**: general cave structure;
- **Research_History**: history of cave research;
- **Notes**: miscellaneous comments on a cave metadata;
- **Findings**: types of archaeological findings that were documented in the cave;
- **Occupation_Periods**: list of identified periods of human activity in the cave; See Table 2 for the list of periods and their chronology;
- **Lighting_Zone**: light zone ID where logger was placed: Light: area right outside the cave’s main entrance; Twilight: shadowed zone inside the cave; Dark: the place inside a cave where sunlight doesn’t reach at all;
- **Logger_Serial_Number**: logger’s manufacture serial number;
- **Logger_Longitude**: logger’s longitude (WGS84) [degrees east];
- **Logger_Latitude**: logger’s (WGS84) [degrees north];

## Technical Validation

The temperature measurement data were visually inspected using the *ICCP* application^51^. The ‘Zones’ mode was selected, and the *CHELSA* daily temperature was plotted against the temperatures recorded in different zones of each cave. A “smooth” curve was added to the plot to represent the general temperature trend. In this way, cave by cave, we visually verified that our logger-based temperature measurements corresponded with the *CHELSA* data, both in overall trends and in distinct short-term fluctuations. Humidity recordings also looked consistent with seasonal dynamics and *CHELSA* precipitation data.

It is worth mentioning a timestamp issue revealed during data compilation: due to the winter–summer time shift occurring at the end of March, each logger recordset contained a single duplicated timestamp. These duplicates were manually corrected.

## Usage Notes

**Fig. 5:**
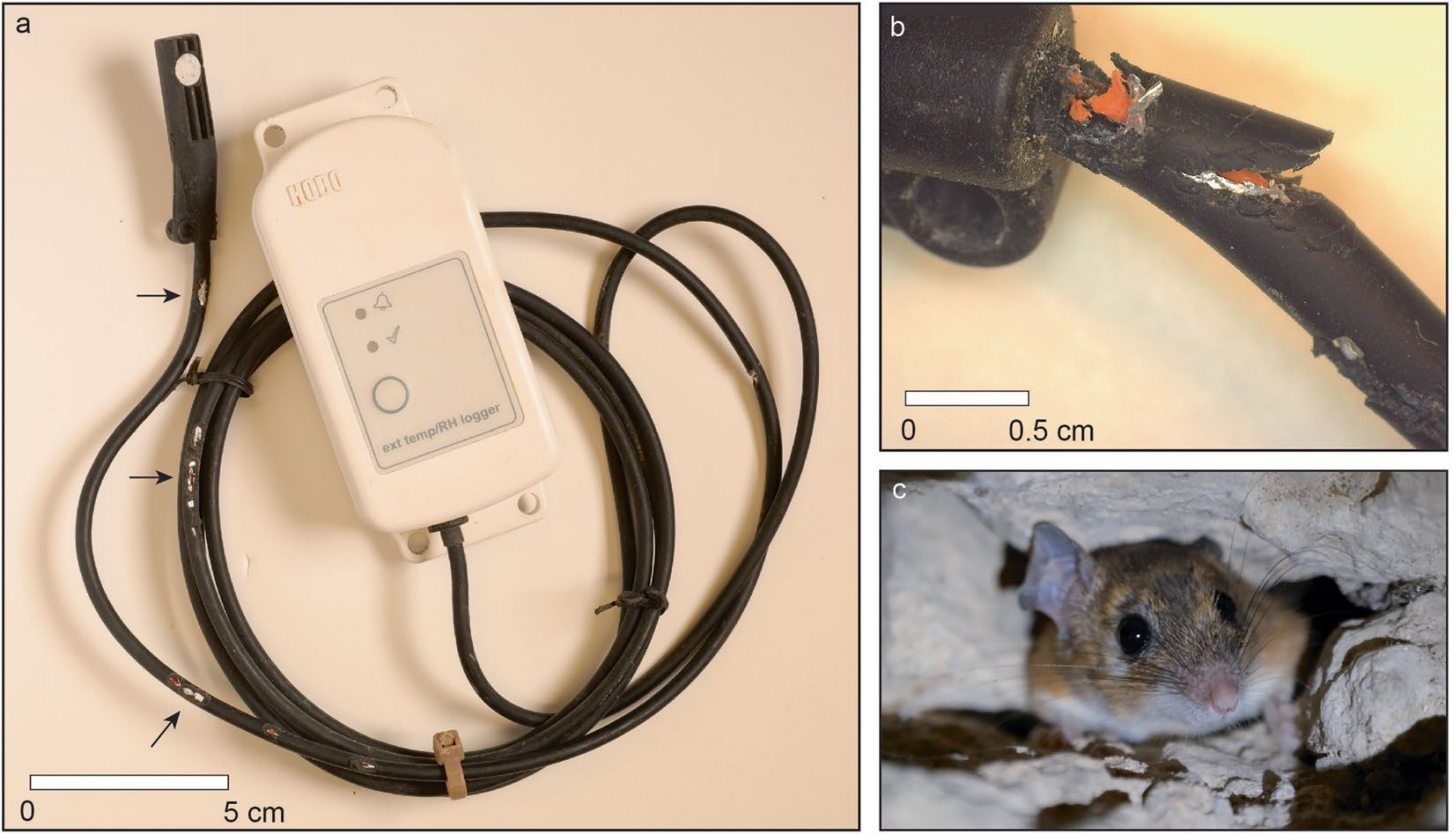
Damage to a logger caused by rodent gnawing: (**a**) a logger with its cable partially gnawed in several places (indicated by black arrows); (**b**) the cable at the connection point to the sensor, severely gnawed—note the teeth marks; (**c**) a spiny mouse in a cave (photos a–b by R. Shafir; photo c by BL).

Out of 44 installed loggers, 42 were retrieved. Despite our efforts to hide and camouflage the loggers, the control (light zone) loggers disappeared from Hemar (logger #3) and Qina (logger #4) caves. It is most likely that curious visitors took these loggers. Out of 42 retrieved loggers, four stopped recording soon after installation, or significantly later, due to rodent activity, mostly by gnawing the cable connecting the sensor to the logger unit. Rodent tooth marks were clearly visible on many logger wires. When rodents not only damaged the plastic wire insulation but also cut through the metal wire, signal transmission was interrupted, and no more data were recorded (Fig. 5). Four loggers were disabled this way: one in the Pool cave, two in Pitriya cave and one in Qina cave.

## Data Availability

The dataset is available at Zenodo (10.5281/ZENODO.17505739)^51^.

## Code availability

While the netCDF data file^51^ can be operated and managed in any suitable software, we also offer the Shiny-based *ICCP* package built for *R* v. 4.5.2, that we wrote to overview the data file along with the comparative *CHELSA* data. The code is open-source and can be found either on Zenodo^51^, or GitHub repository.
